# An Automatic Gastrointestinal Polyp Detection System in Video Endoscopy Using Fusion of Color Wavelet and Convolutional Neural Network Features

**DOI:** 10.1155/2017/9545920

**Published:** 2017-08-14

**Authors:** Mustain Billah, Sajjad Waheed, Mohammad Motiur Rahman

**Affiliations:** ^1^Department of Information and Communication Technology, Mawlana Bhashani Science and Technology University, Tangail, Bangladesh; ^2^Department of Computer Science and Engineering, Mawlana Bhashani Science and Technology University, Tangail, Bangladesh

## Abstract

Gastrointestinal polyps are considered to be the precursors of cancer development in most of the cases. Therefore, early detection and removal of polyps can reduce the possibility of cancer. Video endoscopy is the most used diagnostic modality for gastrointestinal polyps. But, because it is an operator dependent procedure, several human factors can lead to misdetection of polyps. Computer aided polyp detection can reduce polyp miss detection rate and assists doctors in finding the most important regions to pay attention to. In this paper, an automatic system has been proposed as a support to gastrointestinal polyp detection. This system captures the video streams from endoscopic video and, in the output, it shows the identified polyps. Color wavelet (CW) features and convolutional neural network (CNN) features of video frames are extracted and combined together which are used to train a linear support vector machine (SVM). Evaluations on standard public databases show that the proposed system outperforms the state-of-the-art methods, gaining accuracy of 98.65%, sensitivity of 98.79%, and specificity of 98.52%.

## 1. Introduction

The most leading cause of death in the whole world is cancer. Again, gastrointestinal cancer is the most commonly occurring cancer which originates from gastrointestinal polyps. Actually, gastrointestinal polyps are the abnormal growth of tissue on gastric and colonic mucosa. This growth is a slow process and in majority of the cases, before reaching a large size, they do not produce symptoms. However, cancer is preventable and curable, if polyps could be detected early.

Video endoscopy is the most used diagnostic modality for gastrointestinal polyps. In typical video endoscopy, a small camera is entered and directed through the gastrointestinal tract to detect and remove polyps. But typical video endoscopy takes long period of time generating a long video. So, as an operator dependent procedure, it is not possible for a medical person to examine it with sufficient attentiveness during such long and back-to-back endoscopy. However, accuracy of the diagnosis depends on doctor's experience. So, in the examination, some polyps may be undetected. This misdetection of polyps can lead to malignant tumors in the future. Computer aided polyp detection system can reduce polyp misdetection rate and assists doctors in finding the most important regions to be analyzed. Such system can support diagnosis procedure by detecting polyps, classifying polyps, and generating detailed report about any part that should be examined with more attention. Again, duration of this uncomfortable process for the patients and the cost of operation can also be reduced.

A large number of methods have been proposed and applied for computer aided polyp detection system. Covariances of the second-order statistical measures over the wavelet frame transformation (CWC) of different color bands have been used as the image features in [1] for colonoscopy tumor detection with 97% specificity and 90% sensitivity. In their consecutive work [2], an intelligent system of SVM and color-texture analysis methodologies was developed having accuracy of 94%. Adaptive neurofuzzy-based approach for polyp detection in video capsule endoscopy (VCE) was proposed by Kodogiannis et al. [3]. Using texture spectrum from different color channels, they obtained 97% sensitivity over 140 images. Alexandre et al. [4] showed the comparison of texture based and color and position based methods performed in database of 4620 images and obtained area under the curve (AUC) value of 94.87% for the texture histogram of RGB + XY. Combination of color and shape features was used to discriminate polyp from normal regions in [5]. About 94.20% accuracy was gained when they used multilayer perceptron (MLP) as the classifier. A deep convolutional neural network based classification problem was studied for classifying digestive organs in wireless capsule endoscopy in [6]. Another computer aided lesion detection system based on convolutional neural network (CNN) is utilized for more features of endoscopy images in [7]. They also showed comparison between CNN features and combination of color histogram features and LBP features in the experiment. Features learned by CNN outperformed the other method. Tajbakhsh et al. presented a new method integrating global geometric constraints of polyp and local patterns of intensity variation across polyp boundaries [8]. In [9], CNN features have been used to improve the accuracy of colonic polyp classification with sensitivity of 95.16% and specificity of 74.19%. A unique 3-way image presentation and convolutional neural network based polyp detection method have been proposed by Tajbakhsh et al. [10]. Jia et al. used 10,000 WCE images for automatic bleeding detection strategy [11]. They also used convolutional neural network (CNN) for this purpose. Ribeiro et al. suggested that features learned by CNN trained from scratch are more relevant for automated polyp detection system [12]. CNN derived features show greater invariance to viewing angles and image quality factors when compared to the Eigen model [13]. However, fusion scheme of wavelet color-texture analysis and convolutional neural network feature has not been reported in the literature to the best of our knowledge.

In this paper, an automatic system has been proposed as a support to gastrointestinal polyp detection. After the endoscopy video is fed into the proposed system, it extracts color wavelet features and convolutional neural network features from each sliding window of video frames. Fusion of all the features is fed into SVM for classifying it as polyp or nonpolyp. Detected polyp window in the frame is marked and showed in the output. Proposed automatic system detects polyps with an accuracy of 98.65%.

The rest of the paper is organised as follows. The proposed system architecture and methods used in the system are described in Section 2. In Section 3, experimental results are analyzed. Finally, the conclusions of this study are presented in Section 4.

## 2. Structure and Methods

Proposed system is implemented in MATLAB 2017a. It takes video endoscopy in different formats such as avi, mp4, and wmv and outputs the characterized video with marked polyps. This system is divided into some segments such as video to preprocessed frame, frame to sliding window, wavelet feature segment, convolution neural network segment, classification segment, and output segment. All the segments and related methods are outlined sequentially (Figure 4).

### 2.1. Video to Preprocessed Frame

**Figure 1:**
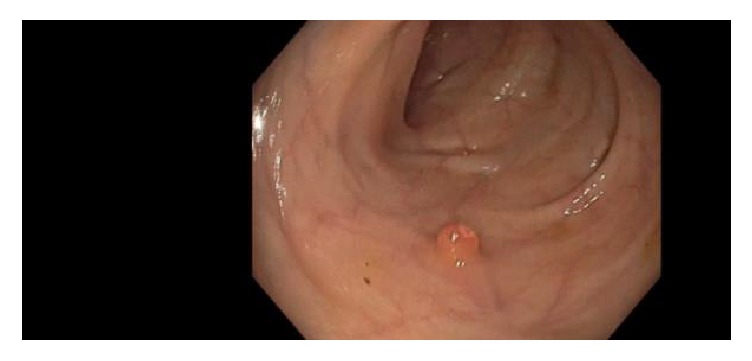
Original video frame.

**Figure 2:**
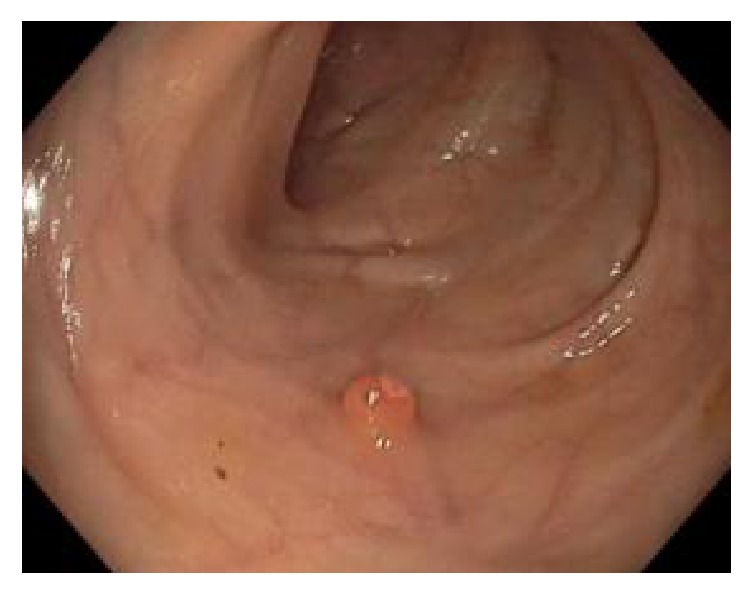
Preprocessed frame.

Endoscopy video to be examined for finding possible polyp is loaded in computer. Then the video is fed into the proposed automatic system. Actually every video is the running sequence of still images called frame. Such a video frame is showed in Figure 2. But all the regions of the original video are not significant, rather there are some unnecessary regions containing description and other information. Examining such regions is nothing but a waste of time. Therefore, unnecessary regions of the original video frame (Figure 1) are discarded resulting in frames as in Figure 2.

### 2.2. Frame to Sliding Window

**Figure 3:**
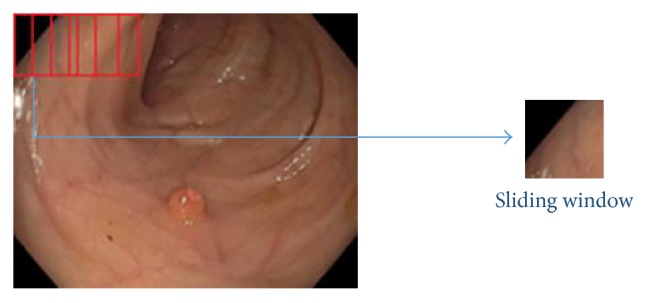
A window of 227*∗*227 is sliding along the frame.

**Figure 4:**
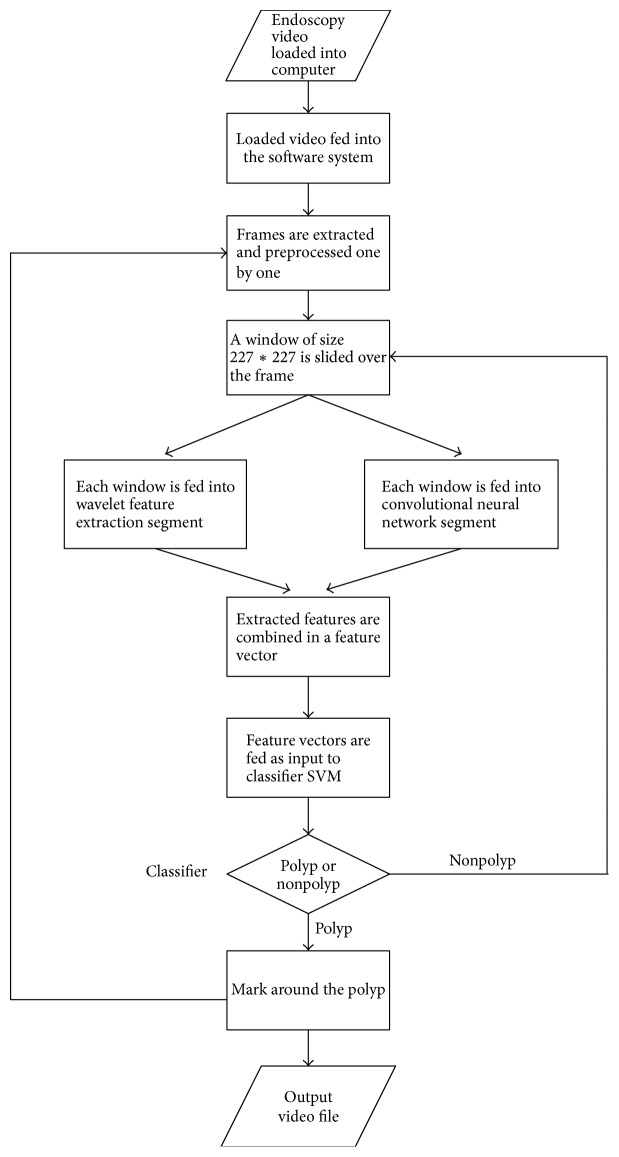
Flow diagram of proposed automatic system.

A window of size 227*∗*227 is slided over the frame from left to right and top to bottom, thus generating small images (called window) from a single video frame as shown in Figure 3. Each window images are considered to be the inputs of feature extraction segment.

### 2.3. Wavelet Feature Segment

The size of polyps varies in different patients. So multiresolutional analysis such as wavelet performs better for textural analysis. But [1] suggests that grayscale textural features are not significant representative for video endoscopy images. So the proposed system uses color textural features from wavelet decomposed images.

Every RGB image has three-color channels: red, green, and blue. So input image, *I* (sliding window) is decomposed into three-color channels *I*^*C*^,* where C* = *r*, *g*, *b*.*D*_6_.

A 3-level and 2-dimensional discrete wavelet transformation is applied on each *I*^*c*^, generating a low resolution image *L*_*CL*_^*C*^ and three-detail image *D*_*CL*_^*C*^, where *CL* = 1,2, 3 …, 9 for 3-level decomposition.

**Figure 5:**
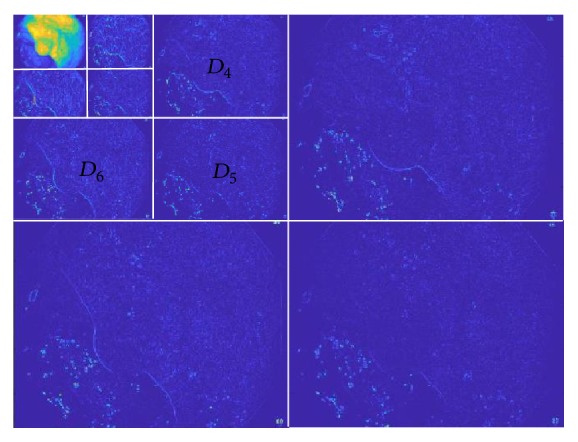
Three-level wavelet decomposition of red channel.

As textural information is localized in the middle wavelet detailed channels original image, only the detail images for *CL* = 4,5, 6 are taken into account (Figure 5). So, finally total nine images {*D*_*CL*_^*C*^} are considered for further processes, where *CL* = 4,5, 6 and *C* = *r*, *g*, *b*.

For finding information about spatial relationships of pixels in an image, another statistical method named cooccurrence matrix is calculated over above nine images. These matrices are calculated in four different directions 0°, 45°, 90°, and 135° generating 36 matrices.

In [14, 15], various statistical features were proposed among which four statistical measures are considered in this proposed system: correlation, energy, homogeneity, and entropy. Finally, four statistical measures for 36 matrices result in total 144 color wavelet features.

### 2.4. Convolution Neural Network Segment

Each window of 227*∗*227 size is inserted into this segment and convolutional neural network features are extracted for the window.

A simple convolutional neural network (CNN) is a sequence of layers where every layer of a CNN transforms one volume of activation to another through a differentiable function. CNNs apply consecutive filters to the raw pixel data of an image to extract and learn different features that can be used for classification. The architecture of a typical CNN is composed of multiple layers where each layer performs a specific function of transforming its input into a useful representation. There are 3 major types of layers that are commonly observed in complex neural network architectures:

- Convolutional layers: in this layer, convolution filters are applied to the image. For each subregion, the layer performs a set of mathematical operations and produces a single value in the output feature map. Convolutional layers then typically apply a ReLU activation function to the output, thus introducing nonlinearities into the model. ReLU, rectified linear unit, is an activation function which can be expressed mathematically: **f**(**x**) = max⁡(0, **x**). A smooth approximation to the rectifier is the analytic function, **f**(**x**) = ln⁡(1 + **e**^**x**^), called the** softplus** function.
- Pooling layers: it downsamples the image data extracted by the convolutional layers and reduces the dimensionality of the feature map to decrease processing time. Max pooling, a commonly used pooling algorithm, extracts subregions of the feature map and discards all other values keeping their maximum value.
- Dense (fully connected) layers: this Layer performs classification on the features extracted by the convolutional layers and downsampled by the pooling layers. In a dense layer, every node in the layer is connected to every node in the preceding layer.

**Figure 6:**
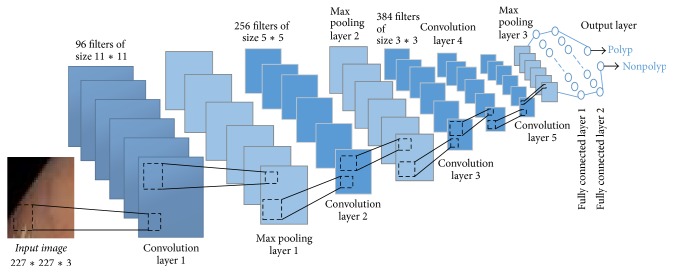
An illustration of the proposed CNN feature extraction segment.

The CNN proposed by this work is inspired by [16]. It contains the following layers, parameters, and configuration (Figure 6):

- Input layer: sliding window image is obtained from video frame of size 227*∗*227*∗*3.
- Two combinations of convolutional and pooling layers: first convolutional layer consists of 96 filters of size 11 × 11 with padding 0 and stride set to 4. The second convolutional layer consists of 256 filters of size 5 × 5 with padding 2 and stride set to 1. Both layers are followed by a ReLU rectifier function. After each convolutional layer, there is a max pooling layer consisting of windows with size of 3 × 3 and stride set to 2.
- Three convolutional layers and a pooling layer: the third, fourth, and fifth convolutional layers are followed by ReLU function containing 384, 384, and 256 filters, respectively. After the three convolutional layers, there is a max pooling layer with size of 3 × 3 and stride set to 2.
- Fully connected layer and the output layer: in a total of three fully connected layers, the first and the second fully connected layers have 4096 neurons each and the third fully connected layer also called output layer has two neurons (polyp and nonpolyp). This output layer can be activated by a softmax regression function.

Each layer of a CNN produces a response, or activation, to an input image. However, there are only a few layers within a CNN that are suitable for image feature extraction. The layers at the beginning of the network capture basic image features, such as edges and blobs. These “primitive” features are then processed by deeper network layers, which combine the early features to form higher level image features. These higher level features are better suited for recognition tasks because they combine all the primitive features into a richer image representation. In this system, features have been extracted from fully connected layer 2.

### 2.5. Classification Segment

Many classifiers have been used for computer aided medical system including linear discriminant analysis (LDA) [1, 17], neural networks [5, 18], adaptive neurofuzzy inference system [3], and support vector machine (SVM) [5, 19]. In this proposed system, SVM has been used for better performance in the case of noisy and sparse data. SVM performance is less affected by feature-to-sample ratio. Many applications have gained better result using SVM for medical image analysis [20, 21].

**Figure 7:**
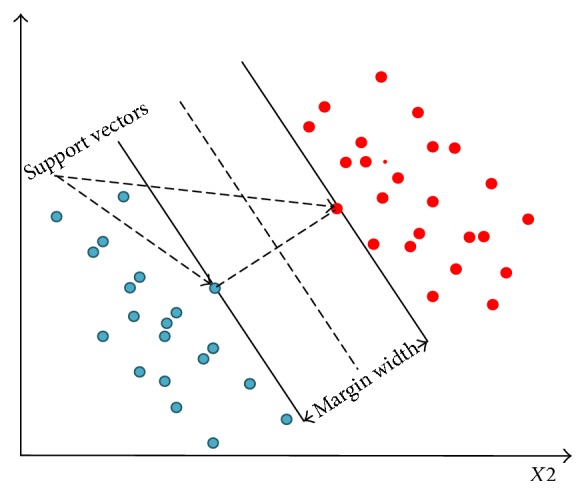
Linear support vector machine.

A support vector machine (SVM) is a binary classifier that tries to find the best hyperplane between data points of two classes. The hyperplane broadens the margin between two classes. The support vectors are the points closest to the hyperplane. An illustration of SVM is given in Figure 7 where blue represents class 1 data points and red represents class 2 data points.

Proposed system launches a multiclass support vector machine using a fast linear solver. From the endoscopy video, polyp and nonpolyp images are extracted. They are split into training and testing datasets. For all the polyp and nonpolyp images, color wavelet and CNN features are extracted. Each image generates 144 color wavelet features and 4096 CNN features which are fused together to form the input feature vector for training SVM classifier.

After the SVM has been trained, it can be used for further polyp and nonpolyp classification tasks. So, using the extracted features of an image window (extracted from frame), classifier gives the decision whether the window is polyp or nonpolyp. If the window is detected as polyp it goes to the output segment; otherwise another consequent window of current video frame comes under feature extraction segment.

### 2.6. Output Segment

**Figure 8:**
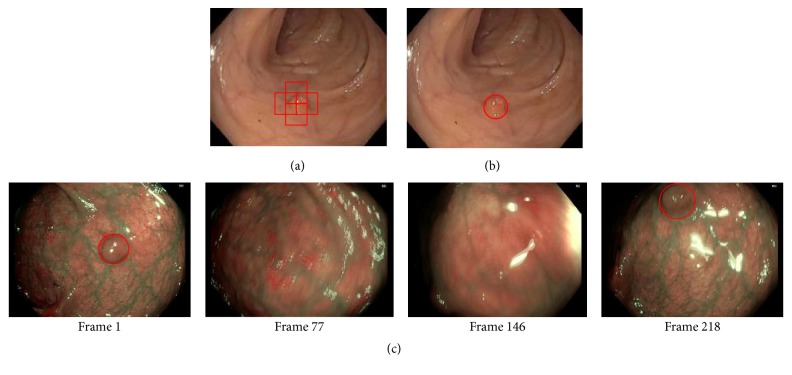
Output segment: (a) several portions are marked to be possible polyp, (b) system's output after processing, and (c) output video frames.

The output of classification segment is processed in this part to mark possible polyp region. As the size of polyps varies in size, different portion of a polyp region may be marked as possible polyp like Figure 8(a). In this situation, score values of each marker region given by SVM are assessed. After the regions with higher scores are found, their positions are averaged to find the final marker as in Figure 8(b). Then the system starts to process the next frame. An illustration of output video frames is shown in Figure 8(c).

## 3. Results

Though feature selection is an important factor for computer aided (CAD) medical video/image data analysis, data availability is another important issue for this purpose. The performance of any CAD depends on training data set. However, the strength of this proposed system is that it utilizes more than 100 standard videos from different sources including its own dataset. Most of the data have been collected from Department of Electronics, University of Alcala (http://www.depeca.uah.es/colonoscopy_dataset/) [16]. Another important source of data set is Endoscopic Vision Challenge (https://polyp.grand-challenge.org/databases/) [22]. Also the proposed system is assessed against standard dataset. Moreover, the proposed system has been tested against human expert's consulted dataset to assess its applicability in real life. From the endoscopy videos, more than 14,000 images are collected for training classifier, among which, one-third of images are polyp and the rest are nonpolyp.

### 3.1. Classifying Different Categories of Polyps

**Figure 9:**
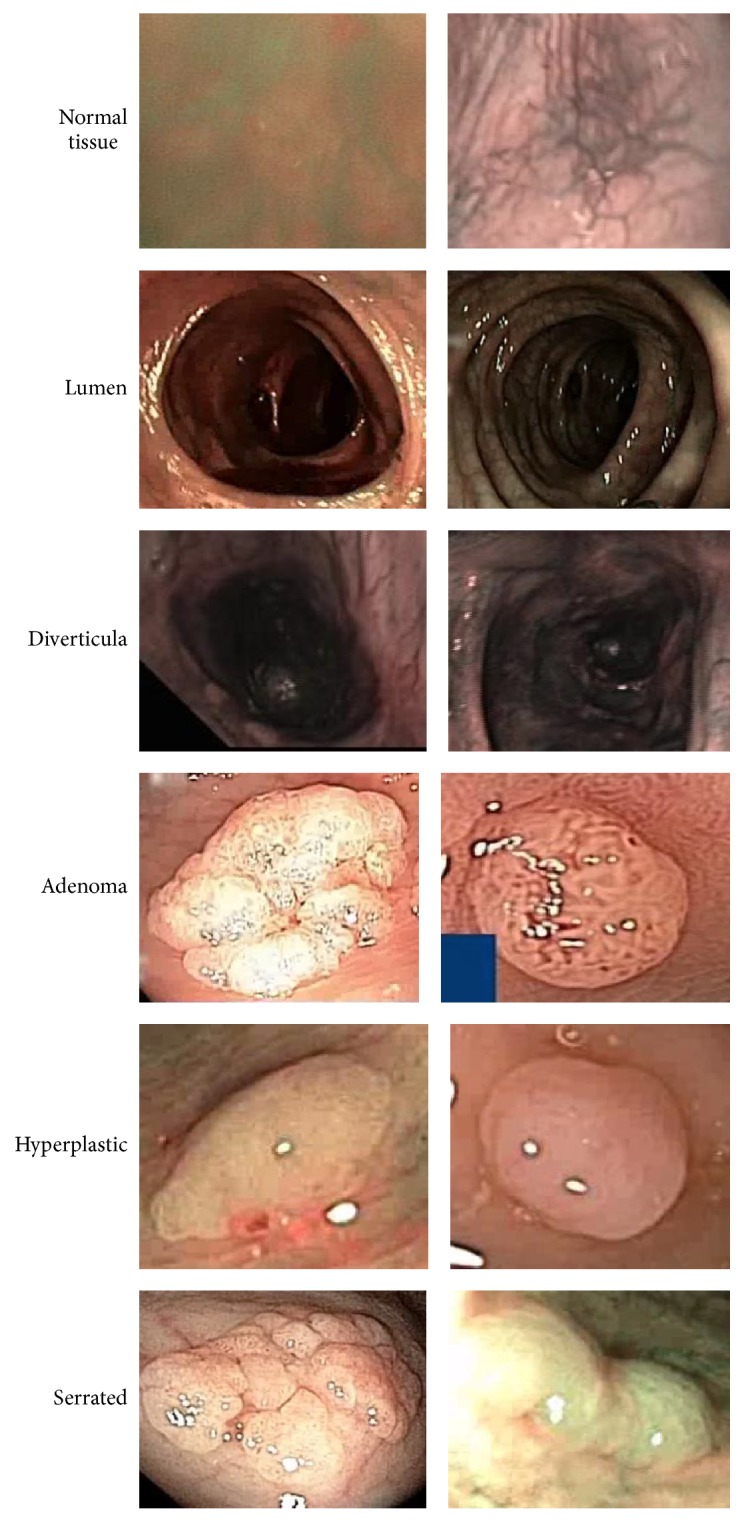
Different categories of video regions and polyps.

Whenever any video is input to the system, it runs a sliding window through the whole regions of the video frame. However, any region may be polyp or nonpolyp. Since there are a number of different categories of polyps, the proposed system is developed in such a way that it can divide the region into different categories such as normal tissue, lumen, diverticula, adenoma, hyperplastic, and serrated polyp. An illustration of different types of video regions is given in Figure 9.

Hyperplastic polyps are large enough and clear, so the proposed system faces no difficulty in identifying hyperplastic polyps. But serrated and adenomas look the same in structure, so sometimes it is difficult to differentiate them. But the proposed system uses convolutional network features, which captures the most primitive and also the higher level features of image, thus easily classifying the video regions. Again lumen and diverticula look similar, but the proposed system captures the deep features of lumen regions, thus identifying them separately. On the other hand, normal tissues have different shapes, sizes, and colors compared with polyp images. So, it may be concluded that the proposed computer aided system can classify a region from a video frame whether it is normal tissue, lumen, and diverticula or hyperplastic, adenomas, and serrated polyps.

### 3.2. Comparison with Other Methods

For evaluating the system, whole dataset is split into training and test dataset. Extracting features from training dataset, support vector machine is trained with those features. Then features from test dataset are extracted and passed through the trained classifier.

For medical data classification, sensitivity (true positive rate) and specificity (true negative rate) are more reliable than accuracy (rate of successful detection). For this system, the following measures are calculated:

(1) Sensitivity=98.79%Specificity=98.52%Accuracy=98.65%.

**Figure 10:**
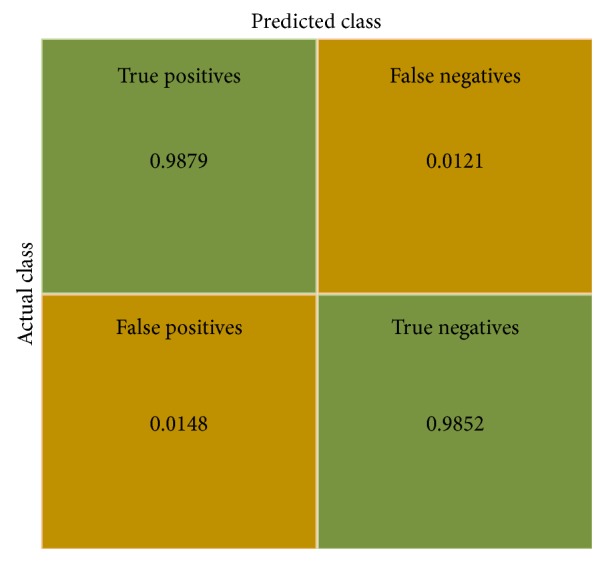
Confusion matrix of the proposed system.

From Figure 10 and information above, it is observed that the proposed fusion model color wavelet features and convolutional neural network features give much satisfactory outcome when choosing SVM as the classifier. A comparison among different polyp detection methods is showed in Table 1.

### 3.3. Comparison with Human Experts

**Figure 11:**
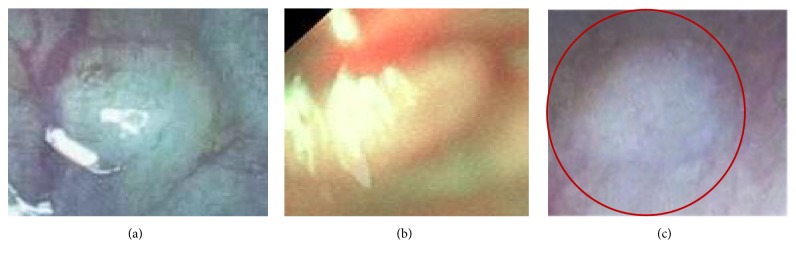
(a) Misdetection by the proposed system. (b) Misdetection by the proposed system. (c) Misdetection by human but correctly detected by the proposed system.

Performance of the proposed approach has been compared with the diagnostic efficacy of human experts. Though nothing can be an alternative to humans, several human factors lead to polyp misclassification. Computer aided polyp detection system not only can assists clinicians, but also can reduce polyp misclassification rate specialty in such cases, where polyps remain undetected for their small sizes. However, to assess the usability of the proposed system, images detected as polyps are fed into the system. Only two images go undetected as polyp images as shown in Figures 11(a) and 11(b). Again, all the test data are used for assessing the system. As the proposed method gains accuracy of 98.65%, results are assessed by medical experts also. They highly appreciate the detection and classification results. Moreover, in some cases, images are detected as polyp which is difficult to detect for human experts also as shown in Figure 11(c).

## 4. Conclusions and Future Works

Computer aided system for automatic polyp detection is of great interest nowadays as a support to the medical persons. Selection of proper features is more important than selection of classifier in automated polyp detection methods. A variety of features have been used for this purpose. In this paper, we have combined the strength of color wavelet features and power of convolutional neural network features. Fusion of these two methodologies and use of support vector machine result in an automated system which takes endoscopic video as input and outputs the video frames with marked polyps. An analysis of ROC reveals that the proposed system may be used for polyp detection purposes with greater accuracy than the state-of-the-art methods. In the future, fusion of CW and CNN features will be used for ultra sound image analysis.

## Figures and Tables

**Table 1:** Comparison among different polyp detection.

Paper	Used methodology	Used dataset	Result		
			Accuracy	Sensitivity	Specificity
Kodogiannis et al. [3]	Texture + ANFIS	140 images		97%	
Park et al. [13]	CNN + CRF	35 videos		86%	85%
Ribeiro et al. [9]	CNN	100 images	90.96%	95.16%	74.19%
Zhu et al. [7]	CNN + SVM	180 images	80%	79%	79.54%
Alexandre et al. [4]	RGB + XY + SVM	4620 images	94.87%		
Zou et al. [6]	DCNN	25 videos	95%		
Li et al. [5]	Color + shape + MLP	450 images	94.20%	95.07%	93.33%
Karkanis et al. [1]	CWC + LDA	60 videos		97%	90%
Iakovidis et al. [2]	KL + wavelet + SVM	86 videos	94%		
*Proposed system*	*Color wavelet + CNN + SVM*	*100 videos*	*98.65%*	*98.79%*	*98.52%*
